# Compound heterozygous mutations in *UBA5* causing early-onset epileptic encephalopathy in two sisters

**DOI:** 10.1186/s12881-017-0466-8

**Published:** 2017-10-02

**Authors:** Gudny A. Arnadottir, Brynjar O. Jensson, Sigurdur E. Marelsson, Gerald Sulem, Asmundur Oddsson, Ragnar P. Kristjansson, Stefania Benonisdottir, Sigurjon A. Gudjonsson, Gisli Masson, Gudmundur A. Thorisson, Jona Saemundsdottir, Olafur Th. Magnusson, Adalbjorg Jonasdottir, Aslaug Jonasdottir, Asgeir Sigurdsson, Daniel F. Gudbjartsson, Unnur Thorsteinsdottir, Reynir Arngrimsson, Patrick Sulem, Kari Stefansson

**Affiliations:** 1 deCODE Genetics/Amgen, Inc., Sturlugata 8, 101 Reykjavik, Iceland; 2 Department of Pediatrics, Landspitali University Hospital, Reykjavik, Iceland; 3 School of Engineering and Natural Sciences, University of Iceland, Reykjavik, Iceland; 4 Faculty of Medicine, University of Iceland, Reykjavik, Iceland; 5 Department of Genetics and Molecular Medicine, Landspitali University Hospital, Reykjavik, Iceland

**Keywords:** Epileptic encephalopathy, *UBA5*, Hypomorphic mutation, Exonic splicing mutation, Ufmylation, Case report

## Abstract

**Background:**

Epileptic encephalopathies are a group of childhood epilepsies that display high phenotypic and genetic heterogeneity. The recent, extensive use of next-generation sequencing has identified a large number of genes in epileptic encephalopathies, including *UBA5* in which biallelic mutations were first described as pathogenic in 2016 (Colin E et al., Am J Hum Genet 99(3):695-703, 2016. Muona M et al., Am J Hum Genet 99(3):683-694, 2016). *UBA5* encodes an activating enzyme for a post-translational modification mechanism known as ufmylation, and is the first gene from the ufmylation pathway that is linked to disease.

**Case presentation:**

We sequenced the genomes of two sisters with early-onset epileptic encephalopathy along with their unaffected parents in an attempt to find a genetic cause for their condition. The sisters, born in 2004 and 2006, presented with infantile spasms at six months of age, which later progressed to recurrent, treatment-resistant seizures. We detected a compound heterozygous genotype in *UBA5* in the sisters, a genotype not seen elsewhere in an Icelandic reference set of 30,067 individuals nor in public databases. One of the mutations, c.684G > A, is a paternally inherited exonic splicing mutation, occuring at the last nucleotide of exon 7 of *UBA5*. The mutation is predicted to disrupt the splice site, resulting in loss-of-function of one allele of *UBA5*. The second mutation is a maternally inherited missense mutation, p.Ala371Thr, previously reported as pathogenic when in compound heterozygosity with a loss-of-function mutation in *UBA5* and is believed to produce a hypomorphic allele. Supportive of this, we have identified three adult Icelanders homozygous for the p.Ala371Thr mutation who show no signs of neurological disease.

**Conclusions:**

We describe compound heterozygous mutations in the *UBA5* gene in two sisters with early-onset epileptic encephalopathy. To our knowledge, this is the first description of mutations in *UBA5* since the initial discovery that pathogenic biallelic variants in the gene cause early-onset epileptic encephalopathy. We further provide confirmatory evidence that p.Ala371Thr is a hypomorphic mutation, by presenting three adult homozygotes who show no signs of neurological disease.

**Electronic supplementary material:**

The online version of this article (10.1186/s12881-017-0466-8) contains supplementary material, which is available to authorized users.

## Background

Epileptic encephalopathies are a group of childhood epilepsies characterized by severe seizures or other paroxysmal electrical activities that contribute to impaired cerebral function [1]. Several causes of epileptic encephalopathies have been described, including cerebral structural abnormalities, inborn errors of metabolism, chromosomal anomalies, and single-gene mutations [1]. A large number of single-gene mutations causing epileptic encephalopathies have been identified through the recent, extensive use of next-generation sequencing [1]. Currently, over 65 genes are known in the early-onset epileptic encephalopathies, of which 20 were discovered in the last three years alone [1, 2].

Here, we describe compound heterozygous mutations in the recently identified epileptic encephalopathy gene, *UBA5*, in two sisters with early-onset epileptic encephalopathy. To our knowledge, this is the first case of biallelic mutations in *UBA5* to be described since the initial reports of pathogenic biallelic variants in *UBA5* causing early-onset epileptic encephalopathy [3, 4]. In addition, we present three adult Icelanders homozygous for one of the mutations detected in the sisters, p.Ala371Thr. The three homozygous individuals show no signs of neurological disease, which confirms that p.Ala371Thr is acting as a hypomorphic mutation.

## Case presentation

**Fig. 1:**
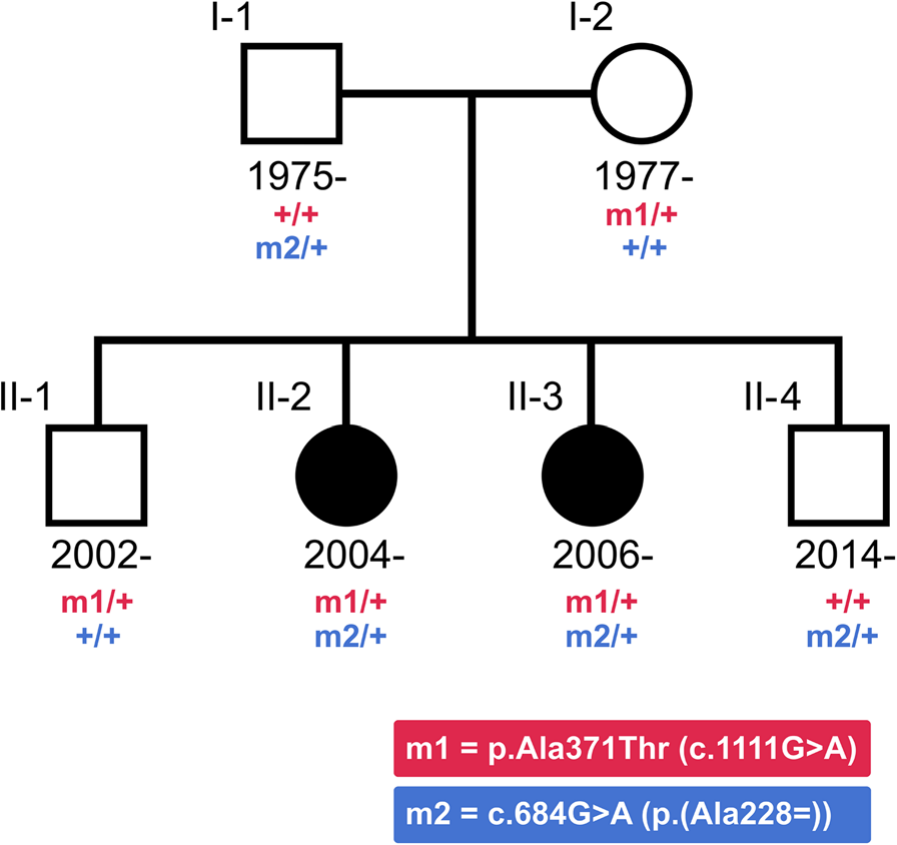
Pedigree of the two affected sisters. Family members are represented by their birth years. The affection status of the sisters is indicated by filled symbols. The status of the two *UBA5* (NM_024818.3) mutations is provided below each family member, where red refers to the status of the missense mutation, p.Ala371Thr (m1), blue refers to the status of the exonic splicing mutation, c.684G > A (m2), and the plus sign (+) refers to the wild-type allele

The two affected sisters were born in 2004 (II-2) and 2006 (II-3) (see pedigree, Fig. 1). Their father (I-1; American, white non-Hispanic, born in 1975), mother (I-2; Icelandic, born in 1977) and two brothers (II-1; born in 2002 and II-4; born in 2014) are unaffected. The pregnancies were uneventful, both sisters were delivered full term with normal birth weight. They first became symptomatic at three months of age, showing failure to thrive and a decline in motor skills such as tracking and head support. The older sister (II-2) was admitted to the Children’s Hospital of Iceland at six months of age because of increasing irritability, nutritional difficulties and episodes of infantile spasms. An electroencephalogram, performed at the time, showed modified hypsarrhythmia. Her seizures initially responded to vigabatrin, the infantile spasms disappeared and she became less irritable. After a few weeks however, the infantile spasms returned and she responded only partly to high doses of adrenocorticotropin. She developed severe intellectual disability with seizures and dystonic cerebral palsy and was diagnosed with early-onset epileptic encephalopathy.

The younger sister (II-3) had a similar clinical course, although slightly milder. She has severe intellectual disability, epilepsy, and quadriplegic cerebral palsy with extrapyramidal features, and received the same diagnosis as her sister of early-onset epileptic encephalopathy. A clinical examination of the two sisters, at ages 10 and 8, showed mild dysmorphic facial features, including a slightly upturned nose and mild hypertelorism, difficulties with visual fixation and following, and low muscle tone (although normal muscle strength proximally and distally). Opthalmologic examinations showed no abnormalities of the retina or optic nerves and normal reactions to strong light, bilaterally. An MRI, performed at ages 12 and 10, showed relatively wide sulci and mild cerebral atrophy (Additional file 1: Figure S1). At their latest examination, both sisters still had frequent seizures. They have shown limited response to several antiepileptic drugs in high doses (including high dose phenytoin, levetiracetam, and high dose vigabatrin). The CARE guidelines were followed in this case description.

In an attempt to find a genetic cause for their condition, we sequenced the genomes of the two sisters and their parents to a targeted depth of 30× (for a detailed description of the whole-genome sequencing see Additional file 2: Supplementary methods). The genetic analysis focused on rare genotypes at coding and splicing regions in the genome (SNPs and small indels). Large-scale sequencing of the Icelandic population by deCODE genetics [5, 6] has yielded a local normative set, currently consisting of 30,067 whole-genome sequenced Icelanders (to a median depth of over 30×). The minor allele frequency of sequence variants was determined from this set of whole-genome sequenced Icelanders as well as from an international dataset of 138,632 individuals either whole-exome or whole-genome sequenced (the genome Aggregation Database, gnomAD [7]). We defined rare autosomal recessive genotypes as homozygous or compound heterozygous sequence variants, each with a minor allele frequency lower than 2% in our set and in gnomAD. We defined rare autosomal dominant genotypes as variants with a minor allele frequency lower than 0.1% in our set and gnomAD. We assessed all possible modes of inheritance but considered a recessive mode of inheritance or a dominant mode of inheritance with parental germline/somatic mosaicism most likely, based on the manifestation of two affected siblings. Due to the extent of clinical similarity and the severity of their condition, we focused on genotypes shared by the two sisters.

We identified two rare genotypes that fulfilled the above criteria (Additional file 3: Figure S2 shows our approach to variant filtering). One of the genotypes was in *MTA1*, a gene not previously known to contribute to Mendelian disease (Additional file 3: Figure S2). The other was a compound heterozygous genotype in *UBA5*, a gene that has only very recently been linked to an epilepsy phenotype [3, 4, 8] (Early Infantile Epileptic Encephalopathy 44, EIEE44, MIM: 617,132, Creation date: 09/27/2016). *UBA5* encodes ubiquitin-like modifier activating enzyme 5, a necessary component of a post-translational modification mechanism known as ufmylation [4]. Compound heterozygous mutations were first reported in *UBA5* in two Chinese siblings presenting with autosomal recessive cerebellar ataxia in February 2016 [8]. In September 2016, two reports describing the pathogenic role of *UBA5* biallelic variants in early-onset epileptic encephalopathy were published [3, 4]. We would like to point to high similarity between the two affected sisters in Iceland and the cases presented in these two reports [3, 4] (Table 1).

**Table 1:** Clinical features of the two affected sisters compared to reported phenotypes in cases of *UBA5 *early-onset epileptic encephalopathy

	Affected sisters	*UBA5* early-onset epileptic encephalopathy cases [2, 3]
Pregnancy and delivery	Normal	Normal
Failure to thrive	Yes	Yes
Central Nervous System	Early-onset epileptic encephalopathy Dystonic cerebral palsy Hypotonia Infantile spasms Seizures: - Recurrent - Resistant to treatment Developmental delay Decline in motor skills	Early-onset epileptic encephalopathy Dystonic movements Spasticity Hypotonia Infantile spasms Seizures: - Recurrent - Pharmaco-resistant Developmental delay Decline in motor skills
Intellectual disability	Severe	Ranging from moderate to severe
Optic fundi	Normal	Normal
Vision	Difficulty in fixating and following	Impaired fixation, squinting
Microcephaly	Yes	Yes (< −3SD), one case with -2SD
Brain MRI	Relatively wide sulci Mild cerebral atrophy	Widening of sylvian fissures (lateral sulcus) Thin corpus callosum White matter hyperintensities Delayed myelination Mild cerebellar hypoplasia Mild cortical and central atrophy Mild cerebral/cerebellar atrophy
EEG abnormalities	Modified hypsarrhythmia	Ranging from none to hypsarrhythmia

The compound heterozygous genotype we detected consists of a paternal exonic splicing mutation, c.684G > A, and a maternal missense mutation, p.Ala371Thr, in *UBA5* (Table 2 and Fig. 1). Sanger sequencing confirmed the genotypes of the sisters and their parents. The two unaffected brothers (II-1 and II-4, Fig. 1 and Additional file 4: Table S1) were Sanger genotyped and each found to be heterozygous for only one of the mutations.

**Table 2:** Annotation of the two *UBA5* mutations detected in the sisters

	mutation A (maternal)	mutation B (paternal)
Position (GRCh38)	chr3:132,675,903	chr3:132,671,881
Variant consequence	Missense	Splice region (donor site) leading to a LoF
Variant genotype	Heterozygous	Heterozygous
cDNA change^a^	c.1111G > A (NM_024818.3)	c.684G > A (NM_024818.3)
Protein change^a^	p.Ala371Thr (NP_079094.1)	p.(Ala228=) (NP_079094.1)
Transcript length	404 AA (NM_024818.3)	404 AA (NM_024818.3)
Exon/exons in transcript	11/12 (NM_024818.3)	7/12 (NM_024818.3)
Allelic frequency Iceland	0.38% (249/30,067 Icelanders)	Absent from 30,064 Icelanders
Allelic frequency abroad (gnomAD [7])	0.58% (75/12,985 Finnish individuals) 0.25% (157/62,175 European, non-Finnish individuals)^b^	Seen in one individual (European, non-Finnish) out of 138,632
SIFT^c^	Deleterious	NA
PolyPhen-2^c^	Possibly damaging	NA
GERP conservation score^d^	Highly conserved (5.44)	Highly conserved (4.95)
Disease in literature	Early Infantile Epileptic Encephalopathy-44 [2–4]	

^a^Functional annotation as suggested by the Human Genome Variation Society (HGVS)

^b^A lower frequency in other populations

^c^SIFT and PolyPhen-2 are scores for the predicted effect of amino acid substitution on protein structure and function

^d^The Genomic Evolutionary Rate Profiling (GERP) framework gives a measure of evolutionary conservation based on the alignment of sequences from 29 mammalian species

The paternally inherited mutation, c.684G > A, is a splice region mutation occurring at the last nucleotide of exon 7 of *UBA5* in the exonic part of the splice donor (5′) region (Additional file 5: Figure S3). It appears to be a very rare and recent mutation, absent from 30,067 whole-genome sequenced Icelanders and detected only once in gnomAD’s set of 138,632 whole-exome/genome sequenced individuals [7]. The mutation affects a highly conserved nucleotide, both in terms of evolutionary conservation (Table 1) and with regards to the consensus sequence for human splice donor regions. Based on both the high conservation of this position and a predicted altered splicing motif (in silico predictions) we suggest that the mutation disrupts the donor splice site of exon 7 of *UBA5*. Two possible scenarios can result from this disruption; A) skipping of the subsequent exon in *UBA5* (exon 8) or multiple exons (exons 8–12) from the mature mRNA or B) a read-through into intron 7 of *UBA5* with the introduction of a premature stop codon 18 amino acids downstream of the exon/intron boundary of exon 7. Both scenarios would result in nonsense-mediated decay of the mature transcript with a corresponding loss-of-function effect. We note that in one of the *UBA5* families described previously (by Muona et al.) a *UBA5* mutation was detected at the third nucleotide of the acceptor (3′) end of exon 2 [4]. That mutation was initially annotated as a missense, p.Arg55His, but was reclassified as a loss-of-function (LoF) mutation when shown to interfere with splicing and result in nonsense-mediated decay [4].

The other mutation detected in *UBA5* in the two sisters is a missense mutation, p.Ala371Thr, inherited from their mother. The mutation has a minor allele frequency of 0.38% in Iceland, 0.58% in Finnish and 0.25% in non-Finnish Europeans, with lower frequencies in other populations [7]. The two reports describing the role of biallelic variants in *UBA5* in early-onset epileptic encephalopathy classify p.Ala371Thr as a hypomorphic allele that contributes to a severe neurological phenotype only when in *trans* with a LoF in *UBA5* [3, 4]. In our set of 150,656 chip-genotyped Icelanders, into which we have imputed all variants detected in a large set of whole-genome sequenced Icelanders [5], we have identified three individuals who are homozygous for the p.Ala371Thr mutation. We have extensive phenotypic information linked to the set of 150,656 genotyped individuals. All three individuals homozygous for p.Ala371Thr have reached adult age without showing signs of a neurological phenotype, and two of them have three and six children, respectively (Table 3). Our findings confirm the hypomorphic nature of p.Ala371Thr and prove that it is only pathogenic when in combination with a LoF in *UBA5*.

**Table 3:** Icelandic individuals homozygous for p.Ala371Thr in *UBA5*

Individual	Gender	Year of birth	Number of offspring	Main reported phenotypes
A	Female	1934-	3	Coronary artery disease Essential hypertension Osteoarthritis
B	Male	1946-	0	Myocardial infarction
C	Female	1940-	6	Coronary artery disease Ulcerative colitis

For the three p.Ala371Thr homozygous individuals detected in the Icelandic reference cohort we show gender, year of birth, number of offspring and main clinical phenotypes. The phenotypes were obtained from the National University Hospital of Iceland’s registry. All three individuals are still alive and two of them have children, none of them has a reported neurological condition. Individuals B and C are siblings

## Discussion and conclusions

We describe a compound heterozygous genotype in *UBA5* in two sisters with early-onset epileptic encephalopathy. The sisters and their parents were initially whole-exome sequenced in 2013. The two compound heterozygous mutations in *UBA5* were detected in the whole-exome data but at the time, no disease-causing mutation had been described in this gene and the genotype was therefore not read as a pathogenic one. We decided to repeat the analysis of the family using whole-genome sequencing in 2016. In September 2016 the two reports [3, 4] on biallelic variants in *UBA5* causing early-onset epileptic encephalopathy were published, allowing us to conclude on the pathogenicity of the compound heterozygous mutations in *UBA5* in the two affected sisters. The progression of this case underlines the importance of periodically revisiting the most recent literature when performing genetic analysis of clinical cases.

We have identified three adult and unaffected individuals homozygous for one of the mutations detected in the two sisters, p.Ala371Thr, thus confirming that it is hypomorphic and not disease-causing by itself. As we do, the authors of the two reports on biallelic variants in *UBA5* describe the combination of p.Ala371Thr and a loss-of-function mutation to cause early-onset epileptic encephalopathy [3, 4]. The paternally inherited exonic splicing mutation we present is predicted to result in abnormal splicing of *UBA5* with a subsequent loss-of-function effect.

*UBA5* is the first ufmylation gene identified in epileptic encephalopathies. Although a link between defects in other post-translational modification mechanisms and this disease group has been demonstrated [9], the exact role of impaired ufmylation in epileptic encephalopathy is still unclear. The identification and reporting of new pathogenic *UBA5* genotypes adds necessary evidence to the pathogenetic story of *UBA5* and defects in the ufmylation process.

## Additional files

Additional file 1: Figure S1. Brain MRI of the older sister. Axial T2 image of the older sister (II-2) showing relatively wide sulci and a mild cerebral atrophy. (DOCX 21 kb)

Additional file 2: Supplementary methods. A detailed description of the sample acquisition, sample preparations, whole-genome sequencing, alignment, variant calling and annotation. (DOCX 148 kb)

Additional file 3: Figure S2. Variant filtering flowchart. This flowchart depicts how we narrowed nearly 5,000,000 variants from the sequenced genome of each affected sister down to a single shared genotype; compound heterozygous mutations in the gene *UBA5*. (DOCX 71 kb)

Additional file 4: Table S1. Sequencing data metrics for the *UBA5* missense mutation (p.Ala371Thr) and exonic splice (c.684G > A). From whole-genome sequencing (WGS) and whole-exome sequencing (WES) data: Call ratios of the alternative alleles in sequenced family members (the two sisters and their parents) and the sequencing depths of the reference and alternative alleles of the two mutations. Also shown are results from the Sanger sequencing of the two mutations in all family members, including the two unaffected brothers (II-1 and II-4) who were neither whole-exome nor whole-genome sequenced. (DOCX 87 kb)

Additional file 5: Figure S3. *UBA5* (NM_024818.3). The positions of the exonic splicing mutation (c.684G > A) and the missense mutation (p.Ala371Thr). (DOCX 14 kb)

## Abbreviations

EEG: Electroencephalogram
EIEE44: Early infantile epileptic encephalopathy 44
gnomAD: Genome aggregation database
LoF: Loss of function
MIM: Mendelian inheritance in man
MRI: Magnetic resonance imaging
mRNA: Messenger ribonucleic acid
MTA1: Metastasis associated 1
SNPs: Single nucleotide polymorphisms
UBA5: Ubiquitin like modifier activating enzyme 5
