# Supplementary material for: Compound heterozygous mutations in UBA5 causing early-onset epileptic encephalopathy in two sisters
Source: BMC Med Genet. 2017 Oct 2;18:103. doi: 10.1186/s12881-017-0466-8 (PMC5623963; doi:10.1186/s12881-017-0466-8)
Supplement: Supplementary file 1 — Brain MRI of the older sister. Axial T2 image of the older sister (II-2) showing relatively wide sulci and a mild cerebral atrophy. (DOCX 21 kb) [file 12881_2017_466_MOESM1_ESM.docx]

|  |  |
| --- | --- |
| **Figure S1.** **Brain MRI of the older sister.** Axial T2 image of the older sister (II-2) showing relatively wide sulci and a mild cerebral atrophy. | |
