# Supplementary material for: Compound heterozygous mutations in UBA5 causing early-onset epileptic encephalopathy in two sisters
Source: BMC Med Genet. 2017 Oct 2;18:103. doi: 10.1186/s12881-017-0466-8 (PMC5623963; doi:10.1186/s12881-017-0466-8)
Supplement: Supplementary file 2 — A detailed description of the sample acquisition, sample preparations, whole-genome sequencing, alignment, variant calling and annotation. (DOCX 148 kb) [file 12881_2017_466_MOESM2_ESM.docx]

**Supplementary methods**

**Sample acquisition and preparation**

Genomic DNA (gDNA) was isolated from blood samples from the two affected sisters and their parents. A request for whole genome sequencing (WGS) was sent through an in-house laboratory information management system (LIMS). gDNA was registered and isolated by an in-house core facility (Biological Materials facility). Samples were delivered to the Genome Sequencing Laboratory in a barcoded 96 well tray format and stored at 4°C until use.

Samples were prepared for sequencing using the TruSeq PCR-free library preparation kits from Illumina. In short, 1 μg of gDNA, isolated from either frozen blood samples or buccal swabs, was fragmented to a mean target size of 300-400 bp using a Covaris E220 instrument. End repair, generating blunt ended fragments was done followed by size selection using different ratios of AMPure XP magnetic purification beads. 3’-Adenylation and ligation of indexed (96 dual indices) sequencing adaptors containing a T nucleotide overhang was performed, followed by AMPure purification.

The quality and concentration of all sequencing libraries is assessed using the LabChip GX (96-samples) instrument from Perkin Elmer. Sequencing libraries are diluted to 3nM concentration and stored at -20 °C. Further quality control of sequencing libraries is done by multiplexing and pooling 96 samples (one 96-well tray) and sequencing each pool on an Illumina MiSeq instrument to assess optimal cluster densities, library insert size, duplication rates and library diversities. All steps in the workflow are monitored using our in-house LIMS, with barcode tracking of all samples and reagents.

**Whole genome sequencing and alignment**

Sequencing libraries are hybridized/clustered to the surface of paired-end (PE) HiSeq X version 2.5 flowcells, using the Illumina cBot™ instrument. Each library is hybridized to a single lane on a flowcell, yielding in general >30× sequence coverage. Paired-end sequencing-by-synthesis (SBS) is performed on Illumina HiSeq X instruments with a readlength of 2×150 cycles of incorporation and imaging. Real-time analysis involves conversion of image data to base-calling in real-time. Basecalling files (BCL) are transferred to an in-house data storage for further secondary pipeline analysis. Monitoring of flowcell/sample registration, run performance/completion and data yield is done via the in-house LIMS.

Sequencing reads were aligned to NCBI's Build 38 of the human reference sequence using the Burrows-Wheeler Aligner (BWA), version 0.7.10 [1]. Alignments were merged into a single BAM file and marked for duplicates using Picard 1.117. Only non-duplicate reads were used for the downstream analyses.

**Variant calling and annotation**

Variants were called using version 2.3-9 of the Genome Analysis Toolkit (GATK) [2], reads were called with GATK’s HaplotypeCaller, version 2014.4-2-g9ad6aa8, using joint calling. Variants were annotated using release 80 of the Variant Effect Predictor (VEP-Ensembl) [3].

We defined rare, autosomal recessive (homozygous and compound heterozygous) genotypes to be composed of two sequence variants, each with a minor allele frequency lower than 2% in Iceland and international databases. We defined rare, autosomal dominant genotypes as variants with a minor allele frequency lower than 0.1% in Iceland and international databases. We assessed *de novo* mutations by comparing the genotypes of the offspring to the parental genotypes, and assessed parental mosaicism based on the allelic ratio of variants in the parents, defining a mosaic genotype as an allelic ratio of 0.3 or lower.

**Supplementary references**

1. Li H, Durbin R. Fast and accurate short read alignment with Burrows-Wheeler transform. Bioinforma Oxf Engl. 2009;25(14):1754-1760.
2. McKenna A, Hanna M, Banks E, et al. The Genome Analysis Toolkit: A MapReduce framework for analyzing next-generation DNA sequencing data. Genome Res. 2010;20(9):1297-1303.
3. McLaren W, Gil L, Hunt SE, et al. The Ensembl Variant Effect Predictor. Genome Biol. 2016;17:122.
