# Supplementary material for: Compound heterozygous mutations in UBA5 causing early-onset epileptic encephalopathy in two sisters
Source: BMC Med Genet. 2017 Oct 2;18:103. doi: 10.1186/s12881-017-0466-8 (PMC5623963; doi:10.1186/s12881-017-0466-8)
Supplement: Supplementary file 3 — Variant filtering flowchart. This flowchart depicts how we narrowed nearly 5,000,000 variants from the sequenced genome of each affected sister down to a single shared genotype; compound heterozygous mutations in the gene UBA5. (DOCX 71 kb) [file 12881_2017_466_MOESM3_ESM.docx]

**
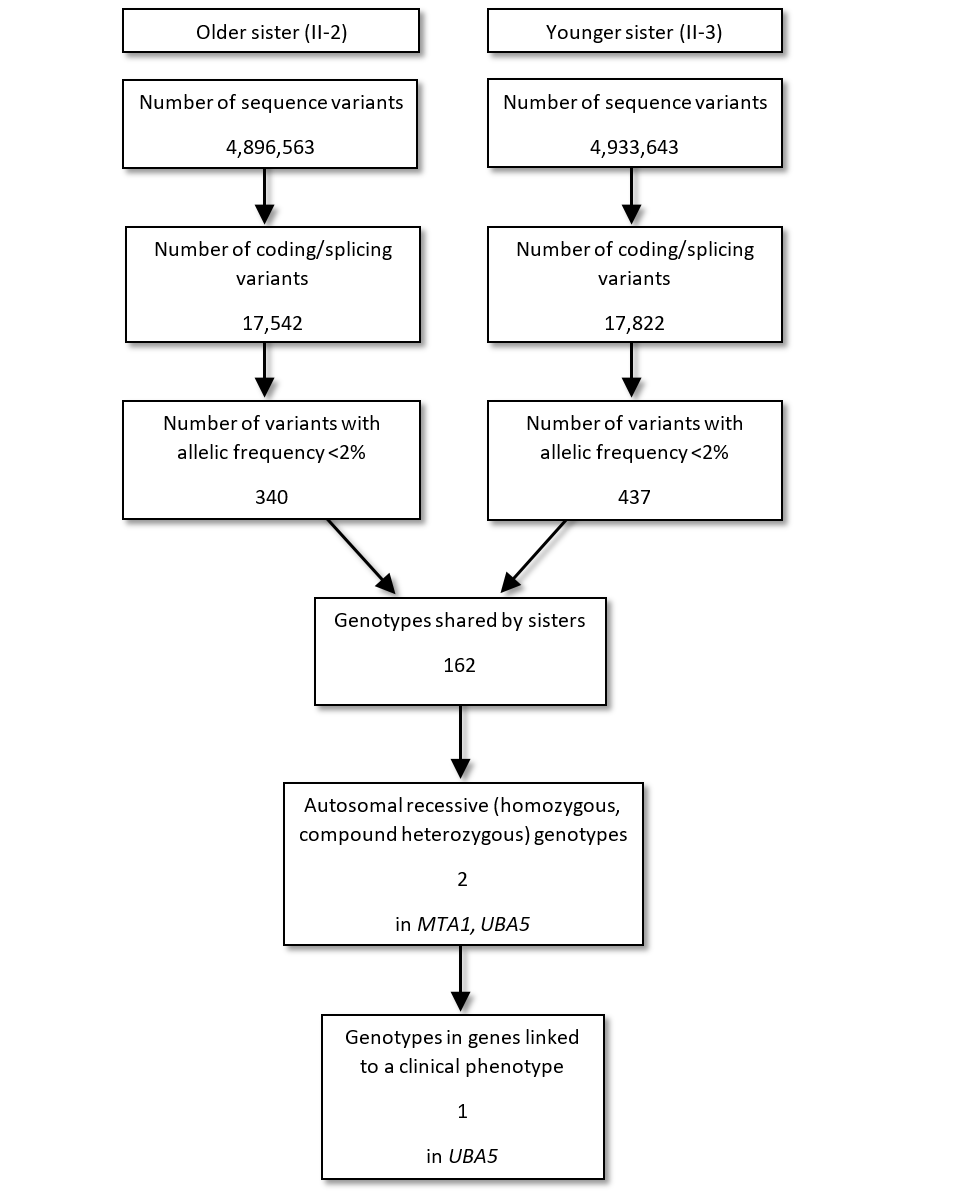
**

**Figure S2.** **Variant filtering flowchart.** This flowchart depicts how we narrowed nearly 5,000,000 variants from the sequenced genome of each affected sister down to a single shared genotype; compound heterozygous mutations in the gene *UBA5*.
