# Supplementary material for: Compound heterozygous mutations in UBA5 causing early-onset epileptic encephalopathy in two sisters
Source: BMC Med Genet. 2017 Oct 2;18:103. doi: 10.1186/s12881-017-0466-8 (PMC5623963; doi:10.1186/s12881-017-0466-8)
Supplement: Supplementary file 4 — Sequencing data metrics for the UBA5 missense mutation (p.Ala371Thr) and exonic splice (c.684G > A). From whole-genome sequencing (WGS) and whole-exome sequencing (WES) data: Call ratios of the alternative alleles in sequenced family members (the two sisters and their parents) and the sequencing depths of the reference and alternative alleles of the two mutations. Also shown are results from the Sanger sequencing of the two mutations in all family members, including the two unaffected brothers (II-1 and II-4) who were neither whole-exome nor whole-genome sequenced. (DOCX 87 kb) [file 12881_2017_466_MOESM4_ESM.docx]

| **Table S1.** **Sequencing data metrics for the *UBA5* missense mutation (p.Ala371Thr) and exonic splice (c.684G>A).**  From whole-genome sequencing (WGS) and whole-exome sequencing (WES) data: Call ratios of the alternative alleles in sequenced family members (the two sisters and their parents) and the sequencing depths of the reference and alternative alleles of the two mutations. Also shown are results from the Sanger sequencing of the two mutations in all family members, including the two unaffected brothers (II-1 and II-4) who were neither whole-exome nor whole-genome sequenced. | | | | | | | | | | | | |
| --- | --- | --- | --- | --- | --- | --- | --- | --- | --- | --- | --- | --- |
| **Family member** |  | **YOB** | **Missense mutation (p.Ala371Thr)** | | | | | Exonic splicing mutation (**c.684G>A)** | | | | |
|  |  |  | **WGS** | | **WES** | | Sanger | WGS | | WES | | Sanger |
|  | **Affection status** |  | **Call ratio** | Ref. / Alt. G / A (x) | **Call ratio** | Ref. / Alt. G / A (x) | Ref. (G)  Alt. (A) | **Call ratio** | Ref. / Alt. G / A (x) | **Call ratio** | Ref. / Alt. G / A (x) | Ref. (G)  Alt. (A) |
| II-2 | A | 2004 | 0.48 | 17 / 16 | 0.61 | 105 / 162 | GA | 0.32 | 30 / 14 | 0.49 | 77 / 75 | GA |
| II-3 | A | 2006 | 0.49 | 20 / 19 | 0.52 | 142 / 156 | GA | 0.48 | 17 / 16 | 0.49 | 70 / 66 | GA |
| I-1 | U | 1975 | 0.0 | 86 / 0 | 0.0 | 258 / 0 | GG | 0.51 | 46 / 47 | 0.58 | 48 / 67 | GA |
| I-2 | U | 1977 | 0.46 | 38 / 33 | 0.54 | 103 / 120 | GA | 0.0 | 93 / 0 | 0.0 | 75 / 0 | GG |
| II-1 | U | 2002 | - | - | - | - | GA | - | - | - | - | GG |
| II-4 | U | 2014 | - | - | - | - | GG | - | - | - | - | GA |
