# Supplementary material for: Compound heterozygous mutations in UBA5 causing early-onset epileptic encephalopathy in two sisters
Source: BMC Med Genet. 2017 Oct 2;18:103. doi: 10.1186/s12881-017-0466-8 (PMC5623963; doi:10.1186/s12881-017-0466-8)
Supplement: Supplementary file 5 — UBA5 (NM_024818.3). The positions of the exonic splicing mutation (c.684G > A) and the missense mutation (p.Ala371Thr). (DOCX 14 kb) [file 12881_2017_466_MOESM5_ESM.docx]

**
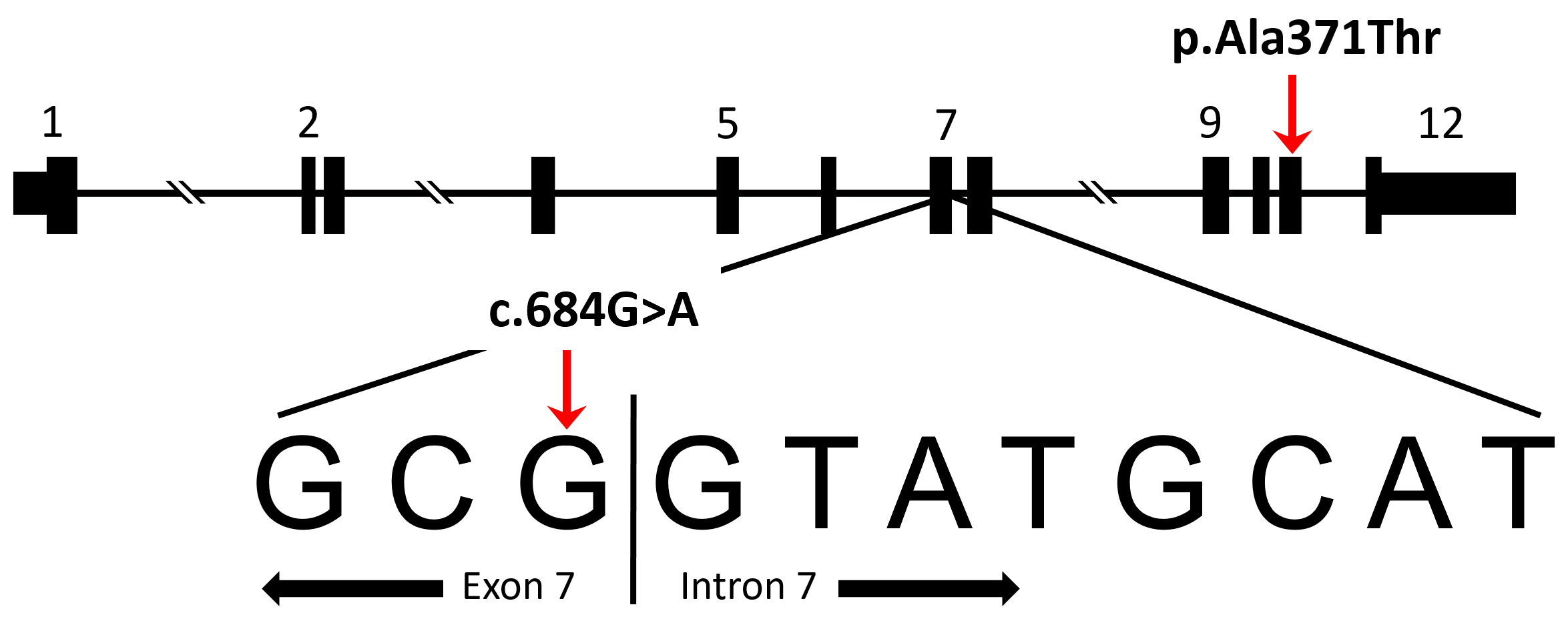
**

**Figure S3**. ***UBA5* (NM_024818.3).** The location of the two mutations detected in *UBA5*, c.684G>A and p.Ala371Thr, shown respective to the exons of the gene. The exonic splicing mutation (c.684G>A) is shown in the context of the splice donor region of exon 7 of *UBA5*, as annotated by Ensembl’s Variant Effect Predictor, i.e. three bases of the exon and eight bases of the intron.
